# Screening Value of Social Frailty and Its Association with Physical Frailty and Disability in Community-Dwelling Older Koreans: Aging Study of PyeongChang Rural Area

**DOI:** 10.3390/ijerph16162809

**Published:** 2019-08-07

**Authors:** Hyungchul Park, Il-Young Jang, Hea yon Lee, Hee-Won Jung, Eunju Lee, Dae Hyun Kim

**Affiliations:** 1 Department of Internal Medicine, Asan Medical Center, University of Ulsan College of Medicine, Seoul 05505, Korea; 2 Pyeongchang Health Center & Country Hospital, Gangwon-do 25377, Korea; 3 Division of geriatrics, Department of Internal Medicine, Seoul National University Bundang Hospital, Seongnam 13620, Korea; 4 Marcus Institute for Aging Research, Hebrew SeniorLife, Boston, MA 02131, USA; 5 Division of Gerontology, Beth Israel Deaconess Medical Center, Boston, MA 02215, USA

**Keywords:** social frailty, physical frailty, disability, geriatric assessment, public health practice

## Abstract

Population aging is a challenge, therefore efficient frailty screening has been increasingly emphasized for mass older populations. This study aimed to evaluate the prevalence of social frailty and its association with physical frailty, geriatric syndromes and activity of daily living (ADL) disability in community-dwelling older adults. A cross-sectional study was conducted with 408 older adults (mean age, 75 years; 58% female) in the Aging Study of PyeongChang Rural Area. A five-item social frailty index was administered (range: 0–5); (1) going out less frequently; (2) rarely visiting the homes of friends; (3) feeling unhelpful to friends and family; (4) being alone; and (5) not talking with someone every day. Social frailty was defined as ≥2 positive responses. Physical frailty was assessed according to the Cardiovascular Health Study frailty phenotype criteria. We used logistic regression to examine whether social frailty can identify older adults with common geriatric syndromes including ADL disability, independently of age, gender, and physical frailty. Social frailty was present in 20.5% (14.5% in male and 25.0% in female) and 11.5% was not overlapped with physical frailty. Social frailty increased risk of ADL disability (odds ratio, 2.53; 95% confidence interval, 1.26–5.09) and depressed mood (odds ratio, 4.01; 95% confidence interval, 1.30–12.39) independently of age, gender, and physical frailty. The predictive power for disability was maximized by using both frailty indices (C statistic 0.73) compared with either frailty index alone (C statistic: 0.71 for social frailty and 0.68 for physical frailty). Social frailty screening is important as it can identify frail older adults who are not captured by demographic characteristics and physical frailty. Moreover, assessment of both social frailty and physical frailty can better detect disability and geriatric syndromes.

## 1. Introduction

Frailty in older adults is defined as a decreased physiological reserve associated with increased vulnerability to various stressors [1]. In Korea 10–16% are frail and 43–59% are prefrail in an urban population [2]. On the other hand, older Koreans living in rural communities have a disproportionately greater burden of frailty and aging-related health conditions than those in urban communities. One study showed that 17.4% were frail and 52.6% were prefrail in a rural population [3].

Although now it is recognized that the concept of frailty consists of physical, psychological, and social domains [4,5], only the physical frailty domain has been studied dominantly in the past. The importance of social frailty has been emphasized only recently. Bunt et al. proposed that social frailty is a continuum of being at risk of losing, or having lost general or social resources, social behaviors and activities, and self-management abilities [6]. Social frailty is a serious concern for aged societies as it can be shown to associate with future disability, developing physical frailty, muscle weakness, cognitive impairment and mortality [7,8,9,10,11,12,13,14,15,16]. 

Recently, various social frailty assessment tools have been developed by several researchers [8,9,17]. These tools are easy to use by non-healthcare professionals in community settings, but are not yet well established and how social frailty and physical frailty differ in their ability to detect disability is not fully understood. Moreover, social frailty in rural areas have not been fully evaluated. 

The objective of this study is to evaluate the prevalence of social frailty and its association with physical frailty, geriatric syndromes, and disability in community-dwelling older adults in rural area. A better understanding of the association between social frailty and physical frailty in rural populations would allow the refinement of community-based interventions to prevent disability in older adults. 

We hypothesize that in resource-limited rural settings, assessment of social frailty will show a high prevalence and that assessment of both social frailty and physical frailty in community-dwelling adults can better detect disability and geriatric syndromes compared to social frailty or physical frailty assessment alone.

## 2. Materials and Methods

### 2.1. Study Design and Sample

The Aging Study of PyeongChang Rural Area (ASPRA) is a population-based cohort study for frailty and geriatric syndromes in older adults living in a rural area. Detailed information on study design, target population, and measurements in ASPRA were described previously [3,18]. Participants who are living in PyeongChang County, Gangwon Province, Korea, were enrolled and annually administered comprehensive geriatric assessments including medical, physical, and psychosocial status. The inclusion criteria were (1) age ≥ 65 years; (2) registered in the National Healthcare Service; (3) ambulatory with or without an assistive device; (4) living at home; (5) able to provide informed consent. The exclusion criteria were (1) living in a nursing home; (2) hospitalized; or (3) bed-ridden and receiving nursing-home-level care at home at the time of enrollment. Of the total of 410 participants in ASPRA cohort, 2 participants were excluded due to incomplete physical function assessment and we cross-sectionally analyzed the data from 408 participants who completed the comprehensive geriatric assessment between 1 July 2018 and 31 August 2018. The Institutional Review board of Asan Medical Center, Seoul, Korea, approved the protocol for this study. Written informed consent was obtained from all participants.

### 2.2. Assessment of Social Frailty

The 5-item social frailty questionnaire by Makizako et al. [8] and Tsutsumimoto et al. [7] were administered in this study: (1) “Do you go out less frequently compared with last year?”; (2) “Do you sometimes visit your friends?”; (3) “Do you feel you are helpful to friends or family?”; (4) “Do you live alone?”; and (5) “Do you talk with someone every day?”. Answers of “yes” to the questions 1, 4 and “no” to the questions 2, 3, and 5 were considered as negative responses. Based on the total number of negative responses, individuals were classified according to the priori determined cut-points as robust if the score was 0, prefrail if 1, and frail if 2–5.

### 2.3. Assessment of Physical Frailty

The Cardiovascular Health Study (CHS) scale was used to measure physical frailty [19]. This scale assigns 1 point to each of the following five components: (1) exhaustion (“moderate or most of the time during the past week” to either of the following statements, “I felt that everything I did was an effort” or “I could not get going”); (2) low activity (lowest quintile in physical activity level measured using the International Physical Activity Questionnaire Short Form [18,20]); (3) slowness (usual gait speed <0.8 m/s from the 4-m walk test); (4) weakness (dominant hand grip strength <26 kg for men and <17 kg for women); and (5) weight loss (unintentional weight loss >3 kg during the previous 6 months). Individuals were classified as robust if the score was 0, prefrail if 1–2, and frail if 3–5. 

### 2.4. Assessment of Geriatric Conditions

Trained nurses and study clinicians administered a comprehensive geriatric assessment. Activities of daily living (ADL) disability was defined as requiring assistance in carrying out any of bathing, continence, dressing, eating, toileting, transferring, and washing face and hands [11]. Multimorbidity was defined as having 2 or more of the 11 physician-diagnosed conditions, including angina, arthritis, asthma, cancer, chronic lung disease, heart failure, diabetes mellitus, heart attack, hypertension, kidney disease, and stroke. Cognitive impairment was determined if the score on the Korean version of the Mini-Mental State Examination was lower than 24 points [21,22]. Depressed mood was determined if the score on the Korean version of the Center for Epidemiological Studies Depression scale was ≥21 points [3,23]. Sarcopenia was determined by the consensus algorithm from the Asian Working Group for Sarcopenia [24]. Dysmobility was defined as a usual gait speed <0.6 m/s from a timed 4-m walk [25]. Polypharmacy was defined as taking 5 or more prescription medications. Malnutrition was defined as Mini-Nutritional Assessment-Short Form (MNA-SF) score ≤11 points [26]. We asked if there was an event of fall during the past year. 

### 2.5. Statistical Analysis

We described the prevalence of social frailty, the social frailty questionnaire itemized responses in study participants and their difference between genders. The characteristics of study participants were summarized by the mean and standard deviation (SD) for continuous variables and proportions for categorical variables. To evaluate the association of social frailty and physical frailty, the proportion of participants with social frailty and physical frailty was visualized with Venn diagram, and we examined the prevalence of common geriatric syndromes for those who had social frailty or physical frailty. Logistical regression was used to estimate the odds ratio (OR) and 95% confidence interval (CI) for each geriatric syndrome associated with both social and physical frailty, social frailty alone, physical frailty alone, or neither frailty after adjusting for age and gender. To evaluate the relationship between each components of social frailty questionnaire according to the CHS scale, Spearman’s rho was applied. We performed the receiver operating characteristic (ROC) analyses and compared C statistics for social frailty (continuous scale), physical frailty (continuous scale), and both frailty scales combined (continuous scale) to compare the abilities in predicting ADL disability. Maximum sensitivity, specificity and cut point were evaluated based on Youden index. Statistical analyses were performed using SPSS version 21.0 (IBM Corporation, Armonk, NY, USA). A two-sided *p*-value < 0.05 was considered statistically significant.

## 3. Results

### 3.1. Psychometrics of Social Frailty

#### 3.1.1. Content Validity

The social frailty score (0 to 5) was positively associated with the CHS score (0 to 5) (rho = 0.420; *p* < 0.001). Social frailty score correlated with most geriatric syndromes including multimorbidity, cognitive impairment, depressive mood, sarcopenia, dysmobility, fall, polypharmacy and malnutrition (rho range: 0.172 to 0.688, *p* = 0.001 to <0.001) (Table S1).

#### 3.1.2. Construct Validity

The internal consistency and kappa coefficients of social frailty for CHS scale are summarized in Table S2. The kappa coefficients of the four items on the social frailty were statistically significant according to the CHS scale. However, an item asking living alone was not significant (Kappa 0.061, *p* = 0.205).

### 3.2. Prevalence of Social Frailty and Associated Characteristics

**Figure 1:**
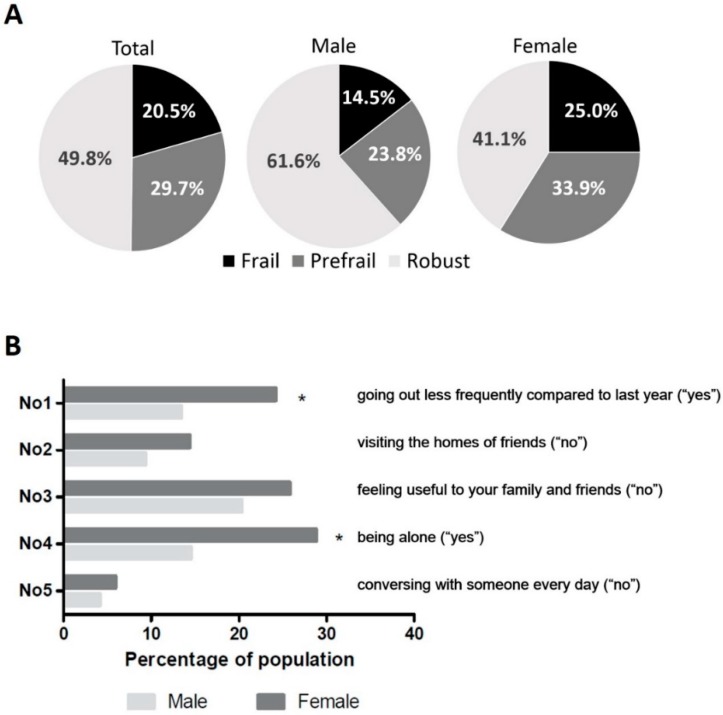
Prevalence of Social Frailty according to gender difference (**A**) and gender difference of Social Frailty questionnaire items (**B**). * statistically significant.

The mean age was 74.9 years (standard deviation 6.0), and 236 (57.8%) were female. The social frailty questionnaire classified 203 (49.8%) as robust, 121 (29.7%) as prefrail, and 84 (20.5%) as frail. The prevalence of social frailty is higher in females (25.0%) than in males (14.5%) (Figure 1A). Of the five social frailty questionnaire items, a large gender difference was observed for two items: women were going out less frequently than men (24.2% vs. 13.4%, *p* = 0.008) and they were more likely to live alone than men (28.8% vs. 14.5%, *p* = 0.001) (Figure 1B).

Table 1 presents clinical characteristics according to the social frailty status. Individuals who were classified frail according to the social frailty questionnaire had older age, higher proportion of women, and greater burden of multimorbidity, cognitive impairment, depressed mood, sarcopenia, dysmobility, fall, polypharmacy, or malnutrition. 

### 3.3. Social Frailty, Physical Frailty, and Geriatric Conditions

**Figure 2:**
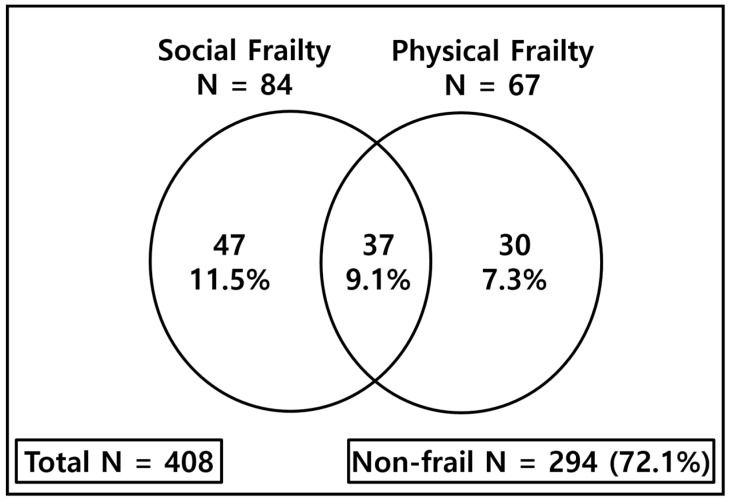
Prevalence of social frailty and physical frailty. N: number.

The prevalence of social frailty is higher than physical frailty (20.6% vs. 16.4%) (Figure 2). The percentage of social frailty alone was 11.5% which was higher than the percentage of physical frailty alone or both social and physical frailty. 

**Figure 3:**
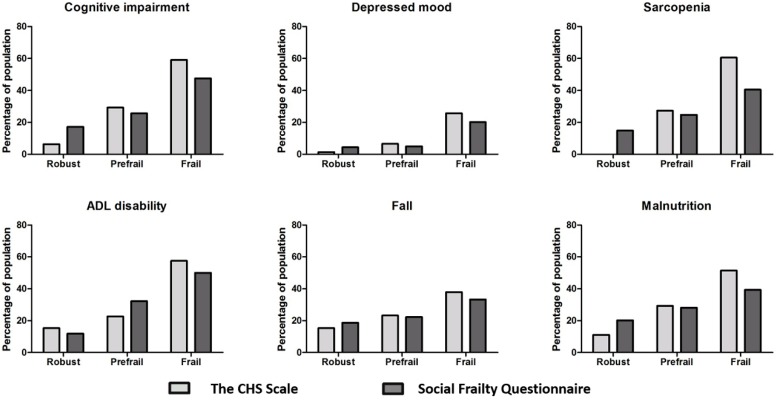
Geriatric syndromes according to social frailty and physical frailty.

The prevalence of geriatric conditions progressively increased from the robust group to the frail group according to both social frailty and physical frailty scales (Figure 3). The CHS scale that measures physical frailty and the social frailty questionnaire that measures social frailty, reflect each other well in detecting various geriatric syndromes in the three groups. 

### 3.4. Association of Social Frailty and Physical Frailty in Geriatric Conditions

Table 2 shows association of social frailty and physical frailty in six geriatric conditions: cognitive impairment, depressive mood, sarcopenia, ADL disability, experience of fall in previous year, and malnutrition. In Model 2 after adjusting for age and gender, social frailty alone independently increased risk of depressive mood (OR, 4.01; 95% CI, 1.30–12.39; *p* = 0.016) and ADL disability (OR, 2.53; 95% CI, 1.26–5.09; *p* = 0.009). Physical frailty alone not only increased risk of depressive mood (OR, 6.41; 95% CI, 1.94–21.14; *p* = 0.002) and ADL disability (OR, 3.10; 95% CI, 1.35–7.14; *p* = 0.008), but also increased risk of sarcopenia (OR, 4.68; 95% CI, 1.95–11.25; *p* = 0.001) and malnutrition (OR, 2.91; 95% CI, 1.29–6.56; *p* = 0.010). However, in the case of cognitive impairment and experience of fall in last year, social frailty alone and physical frailty alone were not independent risk factors. Only both social and physical frailty group showed significantly increased risk of cognitive impairment (OR, 4.48; 95% CI, 1.92-10.44; *p* = 0.001) and experience of fall in previous year (OR, 2.90; 95% CI, 1.30–6.45; *p* = 0.009).

### 3.5. Social Frailty as a Screening Tool of ADL Disability

For prediction of disability, social frailty achieved sensitivity 0.77 and specificity 0.59 at cut-point 1, and physical frailty achieved sensitivity 0.36 and specificity 0.91 at cut-point 3 in our sample. The social frailty showed a C statistic 0.71 (95% CI, 0.65–0.77), which was not statistically significantly different from the physical frailty (C statistic, 0.68; 95% CI, 0.62–0.74) (*p* = 0.36). 

Additionally, social frailty questionnaire and CHS scale were combined into a 10-item combined model and the discriminative function was evaluated. The combined model achieved sensitivity 0.78 and specificity 0.42 at cut-point 2. The discriminatory ability of the combined model (C statistic: 0.73; 95% CI 0.68–0.79) was statistically significantly higher than that of physical frailty (C statistic 0.73 vs. 0.68, *p* = 0.02), but not significantly different from that of social frailty (C statistic 0.73 vs. 0.71, *p* = 0.32).

## 4. Discussion

In our study, we have identified social frailty that cannot be detected by physical frailty. We have confirmed that the predictive power for disability is maximized when we evaluate both social frailty and physical frailty together. 

The prevalence of social frailty was 20.5% in our population of older Koreans living in rural areas, which is considerably more prevalent than urban areas, which reported 10.2% prevalence derived from the identical social frailty assessment tool [8]. This indicates that older adults in rural areas may be more vulnerable to social frailty as there are more aged populations, relatively high prevalence of living alone and low income levels, or lack of accessibility to neighbors and healthcare facilities [3]. However, among five-item social frailty questionnaire, only an item asking living alone was not significant in internal consistency (Table S2). This suggests that living alone may not necessarily induce physical frailty, suggesting that there may be other related causes.

Other studies that used different questionnaires for social frailty reported 18–18.4% prevalence of social frailty among community-dwelling older adults [9,10]. Although the above two studies were conducted with a population that is similar to Makizako et al [8]., the prevalence of social frailty was higher. The major difference is the items of social frailty questionnaire. We used the five-item questionnaire which was found to be associated with a new incidence of need for support and long-term care within 24 months and significantly associated with incident disability [8]. The seven-item questionnaire and four-item questionnaires contain questions about “financial difficulty” that was not included in previous five-item questionnaire. If this study had introduced the questionnaire including the status of finance, the prevalence of social frailty might be higher.

As we see in the previous studies and our study, there is an overlap between social frailty and physical frailty. However, prevalence of social frailty and physical frailty is not fully overlapped. There are vulnerable people who are not evaluated by physical frailty. Our study shows 11.5% of participants have social frailty alone whereas Teo et al. reported 16.8% and Yamada et al. 12.2% of social frailty alone [9,10]. Social activities frequently require higher levels of ability; thus, it has been suggested that social roles may gradually reduce before a decline in cognitive and physical functioning is noted [11]. Therefore, to evaluate the frailty status of society, hidden social frailty needs to be found as it can be the start of a decline in both physical and mental functioning. 

Despite the use of different criteria, previous studies and our study found that both social frailty and physical frailty are associated with increased risk of disability, especially when both frailty criteria are combined [8,9,10]. Additionally, both social frailty and physical frailty increased risk of geriatric conditions, and especially risk of cognitive impairment or fall is increased when social frailty and physical frailty are together. Usually the frailty screening includes physical and psychological components, but not the social components. By the combined use of both physical and social frailty screening, disadvantages of each assessment could be supplemented. 

In detecting disability, social frailty and physical frailty showed similar discriminative power and the combined model of social frailty questionnaire and physical frailty questionnaire showed the highest discriminative power. According to the previous study [9], a combined model showed highest area under the ROC of 0.81 for predicting activities for daily living (ADL) disability, suggesting that a comprehensive evaluation of both social frailty and physical frailty is necessary. We showed that this can be achieved by pooling the five-item social frailty scale and the five-item CHS scale. The major benefits of these scales are that they are self-questionnaires that take less time, do not require trained personnel, and its administration and scoring process is standardized. Therefore these two scales can be easily adapted in public health settings especially in rural areas, as they are time-efficient as well as effective in screening both social and physical frailties. 

Social frailty can be applied to intervention studies that target physical performance or frailty. Many of the recent successful multicomponent trials have provided group exercises rather than home-based individualized exercises [27,28]. Based on the results of this study, we believe that community-based intervention programs that increase social interaction will help improve frailty and geriatric syndromes. Future interventional studies are warranted. 

Our study has strengths and limitations. This population-based cohort study included more than 90% of eligible older adults in the study areas and administered comprehensive and validated assessments of frailty and geriatric conditions with high completion rates. Because our study was conducted in older adults who were living in a rural county in Korea, the prevalence of social frailty might be higher and its association with geriatric syndrome might be stronger than the populations living in urban areas with easy access to health care resources. Our results may not be generalizable to the rural population in western countries. Also, the presence of neurologic disorders such as dementia or Parkinson’s disease which may result in complex physical and social status was not analyzed [29]. Social frailty in the presence of such neurologic disorders needs to be considered and further research is needed. As a cross-sectional study, we were unable to evaluate the association between social frailty and future health outcomes. We anticipate that the ongoing follow-up of ASPRA cohort will provide outcome data in the next 1–2 years.

Our study shows that social frailty, defined using a five-item questionnaire, affects one in every five older Koreans who live in rural communities, and is associated with depressed mood and disability independently of physical frailty. Risk of cognitive impairment and fall were meaningful only when social frailty and physical frailty were considered at the same time. These results suggest the importance of considering both social frailty and physical frailty in evaluation of older adults. Further research is warranted to examine the association of social frailty with future health outcomes, including health care utilization and quality of life, and to benefit from developing interventions that target social frailty domains.

## 5. Conclusions

Our results suggest the importance of screening social frailty in community settings in rural areas as it can identify older adults with poor health status who are not captured by demographic characteristics and physical frailty. Moreover, assessment of both social frailty and physical frailty are important in the prevention or improvement of frail conditions among community-dwelling older adults. 

## Figures and Tables

**Table 1:** Relation of social frailty to geriatric syndromes.

	Social Frailty		
Variables (*n*, %)	Robust	Prefrail	Frail
	N = 203	N = 121	N = 84
Age (mean ± SD)	72.7 ± 5.1	75.4 ± 5.6	79.3 ± 6.2
65–74	123 (60.6)	52 (43.0)	20 (23.8)
75–84	76 (37.4)	61 (50.4)	51 (60.7)
85–	4 (2.0)	8 (6.6)	13 (15.5)
Female, *n*	97 (47.8)	80 (66.1)	59 (70.2)
Multimorbidity, *n*	93 (45.8)	74 (61.2)	64 (76.2)
Cognitive impairment, *n* [21,22]	35 (17.2)	31 (25.6)	40 (47.6)
Depressed mood, *n* [3,23]	9 (4.4)	6 (5.0)	17 (20.2)
Sarcopenia, *n* [24]	30 (14.8)	30 (24.8)	34 (40.5)
Dysmobility, *n* [25]	49 (24.1)	56 (46.3)	59 (70.2)
Fall, *n*	38 (18.7)	27 (22.3)	28 (33.3)
Polypharmacy, *n*	51 (25.1)	40 (33.1)	36 (42.9)
Malnutrition, *n* [26]	41 (20.2)	34 (28.1)	33 (39.3)
Living alone, *n*	2 (1.0)	47 (38.8)	46 (54.8)
Low income, *n*	0 (0)	3 (2.5)	5 (6.0)
The CHS scale, *n* [19]			
Robust	96 (47.3)	37 (30.6)	11 (13.1)
Prefrail	98 (48.3)	64 (52.9)	36 (42.9)
Frail	9 (4.4)	20 (16.5)	37 (44.0)

**Table 2:** Association of social frailty and physical frailty in geriatric syndromes.

Geriatric Conditions	Model 1				Model 2			
	Neither	SF Alone	PF Alone	Both	Neither	SF Alone	PF Alone	Both
**Cognitive impairment**								
Prevalence, number [*n* (%)]	53 (18.0%)	14 (29.8%)	13 (43.3%)	26 (70.3%)	53 (18.0%)	14 (29.8%)	13 (43.3%)	26 (70.3%)
OR (95% CI)	Reference	1.93 (0.97–3.86)	3.48 (1.59–7.59)	10.75 (5.00–23.10)	Reference	1.28 (0.60–2.73)	1.69 (0.71–4.01)	4.94 (2.07–11.79)
*p*-value		0.063	0.002	<0.001		0.53	0.236	<0.001
**Depressed mood**								
Prevalence, number [*n* (%)]	9 (3.1%)	6 (12.8%)	6 (20.0%)	11 (29.7%)	9 (3.1%)	6 (12.8%)	6 (20.0%)	11 (29.7%)
OR (95% CI)	Reference	4.63 (1.57–13.70)	7.92 (2.60–24.11)	13.40 (5.09–35.28)	Reference	4.26 (1.38–13.19)	6.29 (1.91–20.73)	10.66 (3.45–32.93)
*p*-value		0.006	<0.001	<0.001		0.012	0.003	<0.001
**Sarcopenia**								
Prevalence, number [*n* (%)]	42 (14.3%)	12 (25.5%)	18 (60.0%)	22 (59.5%)	42 (14.3%)	12 (25.5%)	18 (60.0%)	22 (59.5%)
OR (95% CI)	Reference	2.06 (0.99–4.28)	9.00 (4.04–20.03)	8.80 (4.23–18.32)	Reference	0.88 (0.37–2.08)	4.71 (1.96–11.31)	3.13 (1.34–7.32)
*p*-value		0.054	<0.001	<0.001		0.763	0.001	0.008
**ADL disability**								
Prevalence, number [*n* (%)]	49 (16.7%)	14 (29.8%)	14 (46.7%)	24 (64.9%)	49 (16.7%)	14 (29.8%)	14 (46.7%)	24 (64.9%)
OR (95% CI)	Reference	3.10 (1.59–6.02)	4.37 (2.00–9.55)	9.23 (4.40–19.38)	Reference	2.54 (1.26–5.13)	3.10 (1.35–7.13)	5.82 (2.56–13.25)
*p*-value		0.001	<0.001	<0.001		0.009	0.008	< 0.001
**Fall**								
Prevalence, number [*n* (%)]	57 (19.4%)	11 (23.4%)	8 (26.7%)	17 (45.9%)	57 (19.4%)	11 (23.4%)	8 (26.7%)	17 (45.9%)
OR (95% CI)	Reference	1.27 (0.61–2.65)	1.51 (0.64–3.57)	3.53 (1.74–7.18)	Reference	1.14 (0.53–2.45)	1.31 (0.53–3.25)	2.85 (1.28–6.36)
*p*-value		0.523	0.346	<0.001		0.745	0.556	0.01
**Malnutrition**								
Prevalence, number [*n* (%)]	61 (20.7%)	13 (27.7%)	14 (46.7%)	20 (54.1%)	61 (20.7%)	13 (27.7%)	14 (46.7%)	20 (54.1%)
OR (95% CI)	Reference	1.46 (0.73–2.94)	3.34 (1.55–7.22)	4.49 (2.22–9.10)	Reference	1.31 (0.63–2.72)	2.92 (1.29–6.59)	3.68 (1.67–8.11)
*p*-value		0.288	0.002	<0.001		0.47	0.010	0.001

Model 1: Unadjusted in the analysis of each geriatric conditions. Model 2: Model 1 plus age, gender and low income. Neither: robust and prefrail groups in both social frailty and physical frailty. SF: frail groups in social frailty only, PF: frail groups in physical frailty only, Both: both frail groups in social frailty and physical frailty, OR: odds ratio, CI: confidence interval.

## Supplementary Materials

The following are available online at https://www.mdpi.com/1660-4601/16/16/2809/s1, Table S1: Validation between social frailty and physical frailty, Table S2: Agreement of social frailty items with CHS scale.
