# Supplementary material for: Screening Value of Social Frailty and Its Association with Physical Frailty and Disability in Community-Dwelling Older Koreans: Aging Study of PyeongChang Rural Area
Source: Int J Environ Res Public Health. 2019 Aug 7;16(16):2809. doi: 10.3390/ijerph16162809 (PMC6720732; doi:10.3390/ijerph16162809)
Supplement: Supplementary file 1 [file ijerph-16-02809-s001.pdf]

Table S1. Validation between social frailty and physical frailty.

	Social frailty					Physical frailty				
**Total sample**	**Robust**	**Prefrail**	**Frail**	***Spearman correlation***	***p*-value**	**Robust**	**Prefrail**	**Frail**	***Spearman correlation***	***p*-value**
	N = 203	N = 121	N = 84			N = 144	N = 198	N = 66		
Age (mean±SD)	72.7±5.1	75.4±5.6	79.3±6.2	0.399	<0.001	71.7±4.3	75.4±5.9	80.3±5.5	0.469	<0.001
Female	97 (47.8%)	80 (66.1%)	59 (70.2%)	0.201	<0.001	63 (43.8%)	121 (61.1%)	52 (78.8%)	0.244	<0.001
Multimorbidity	93 (45.8%)	74 (61.2%)	64 (76.2%)	0.240	<0.001	60 (41.7%)	117 (59.1%)	54 (81.8%)	0.270	<0.001
Cognitive impairment	35 (17.2%)	31 (25.6%)	40 (47.6%)	0.245	<0.001	9 (6.3%)	58 (29.3%)	39 (59.1%)	0.402	<0.001
Depressed mood	9 (4.4%)	6 (5.0%)	17 (20.2%)	0.185	<0.001	2 (1.4%)	13 (6.6%)	17 (25.8%)	0.266	<0.001
Sarcopenia	30 (14.8%)	30 (24.8%)	34 (40.5%)	0.227	<0.001	0 (0%)	54 (27.3%)	40 (60.6%)	0.483	<0.001
Dysmobility	49 (24.1%)	56 (46.3%)	59 (70.2%)	0.364	<0.001	0 (0%)	100 (50.5%)	64 (97.0%)	0.688	<0.001
Fall	38 (18.7%)	27 (22.3%)	28 (33.3%)	0.121	0.014	22 (15.3%)	46 (23.2%)	25 (37.9%)	0.172	0.001
Polypharmacy	51 (25.1%)	40 (33.1%)	36 (42.9%)	0.146	0.003	33 (22.9%)	62 (31.3%)	32 (48.5%)	0.174	<0.001
Malnutrition	41 (20.2%)	34 (28.1%)	33 (39.3%)	0.163	0.001	16 (11.1%)	58 (29.3%)	34 (51.5%)	0.307	<0.001

Table S2. Agreement of social frailty items with CHS scale

Social frailty items		CHS frailty				Sensitivity	Specificity	Accuracy	PPV	NPV	Kappa	*p*-value
		No frailty (N =342)		Frailty (N =66)								
No.1: Do you go out less frequently compared with last year?	No	297	86.8%	31	47.0%	53.0%	86.8%	81.4%	43.8%	90.6%	0.367	<0.001
	Yes	45	13.2%	35	53.0%							
No.2: Do you sometimes visit your friends?	No	317	92.7%	41	62.1%	37.9%	92.7%	83.8%	50.0%	88.6%	0.339	<0.001
	Yes	25	7.3%	25	37.9%							
No.3: Do you feel you are helpful to friends or family?	No	281	82.2%	31	47.0%	53.0%	82.2%	77.5%	36.5%	90.1%	0.297	<0.001
	Yes	61	17.8%	35	53.0%							
No.4: Do you live alone?	No	268	78.4%	47	71.2%	28.8%	78.4%	70.3%	20.4%	85.1%	0.061	0.205
	Yes	74	21.6%	19	28.8%							
No5: Do you talk with someone every day?	No	331	96.8%	56	84.8%	15.2%	96.8%	83.6%	47.6%	85.5%	0.165	<0.001
	Yes	11	3.2%	10	15.2%							

CHS, Cardiovascular Health Study; PPV, positive predictive value; NPV, negative predictive value.
